# *Actinobacillus suis* isolated from diseased pigs are phylogenetically related but harbour different number of toxin gene copies in their genomes

**DOI:** 10.1002/vro2.45

**Published:** 2022-10-03

**Authors:** Dharmasiri Gamage Ruwini Sulochana Kulathunga, Alaa Abou Fakher, Matheus de Oliveira Costa

**Affiliations:** ^1^ Department of Large Animal Clinical Sciences Western College of Veterinary Medicine University of Saskatchewan Saskatoon Saskatchewan Canada; ^2^ Department of Population Health Sciences Faculty of Veterinary Medicine Utrecht University Yalelaan 7 Utrecht The Netherlands

## Abstract

**Objective:**

The Gram‐negative bacterium *Actinobacillus suis* is an agent of global importance to the swine industry and the cause of lethal respiratory or septicaemic disease in pigs of different ages. Between 2018 and 2019, seven commercial farms in western Canada experienced episodes of increased mortality due to *A. suis* infection in grower pigs. The goal of this work was to profile, with molecular methods, *A. suis* isolated from diseased pigs and to compare them to other isolates.

**Design:**

This inferential observational study used nine western Canadian strains obtained from diseased lungs (*n* = 6), heart (*n* = 2) and brain (*n* = 1) and whole genome sequencing was performed. Comparative genomic analyses were performed to characterise the genetic variability, antimicrobial resistance and the virulence genes present.

**Results:**

Compared to the reference strain (ATCC 33415), an increased number of RTX (repeats in the structural toxin) gene copies were identified in strains isolated from organs without a mucosal surface, thus theoretically harder to invade. Western Canadian strains did not harbour genes associated with resistance to antimicrobial agents used in swine production. Novel regions were also identified in the genomes of five of nine strains demonstrating recombination and emergence of novel strains.

**Conclusions:**

The results obtained in this study were associated with the emergence of new lineages. An increased number of RTX toxin gene copies is suggested to be associated with increased virulence. This study will contribute to improve our understanding regarding *A. suis* and may help guide vaccine development and agent control measures.

## INTRODUCTION

*Actinobacillus suis* is a facultative anaerobic, non‐motile, Gram‐negative bacillus. It is an important pathogen of swine, leading to respiratory disease or septicaemia in pigs of different ages and stages of production.^1^, ^2^ The organism was first identified and described in 1955, when it was associated with acute septicaemia and sudden death in domestic swine.^3^ *Actinobacillus suis* colonises the nasopharynx and palatine tonsils of healthy pigs; it is a commensal. It can also colonise the vaginal mucosa of healthy sows. Disease is often associated with stressful conditions such as weaning, farrowing, transportation or suckling.^2^, ^4^ Weaned pigs often do not develop clinical signs and are found dead following fulminant septicaemia, while grower‐finisher or older pigs in high‐health status (HHS) herds develop respiratory signs due to necro‐haemorrhagic pleuropneumonia.^5^, ^6^, ^7^ A third syndrome observed in HHS reproductive‐aged pigs is characterised by acute septicaemia, lethargy, anorexia, fever, abortion and erysipelas‐like lesions on the skin.^6^ Treatment strategies include parenteral antimicrobial therapy with ceftiofur, penicillin, ampicillin, neomycin, gentamicin, sulphadimethoxine, tiamulin, tetracycline or tylosin.^8^ However, variation in sensitivity to antimicrobial therapy has been reported and antimicrobial susceptibility testing is suggested when clinical cases are identified.^8^ Autogenous bacterins have been used to prevent disease and shown variable results.^9^ These evidence a gap in our understanding of *A. suis* pathogenesis, contributing to our inability in developing efficient prevention, treatment and control tools.

*Actinobacillus suis* can be readily isolated using blood and McConkey media, producing β‐haemolytic smooth, translucent colonies.^4^ Genes belonging to different functional classes have been proposed to contribute to virulence and facilitate colonisation of mucosal sites.^10^, ^11^ A previous study described 13 virulence genes in *A. suis*, identified using genome‐wide screening by PCR‐based signature‐tagged transposon mutagenesis.^11^ Noteworthy is the RTX (repeats in the structural toxin) gene family. It encodes a pore‐producing protein and is proposed as a disease determinant associated with *A. suis*. The RTX genes identified in *A. suis* is immunologically and genetically related to the RTX genes described in other pathogenic bacteria such as *Escherichia coli*, *Pasteurella haemolytica* and *Actinobacillus pleuropneumoniae*.^10^

Sporadic outbreaks of *A. suis* were previously reported in Canada, the United States, UK, India, New Zealand, Hungary, Croatia and Australia.^12^, ^13^ Since 1990, an increasing number of clinical disease outbreaks have been reported in pigs of varying ages in North America and considered as an emerging disease.^4^, ^12^, ^14^ Therefore, the purpose of this study was to investigate the molecular epidemiology, virulence and antimicrobial gene profile in *A. suis* isolated from diseased pigs in western Canada.

## MATERIAL AND METHODS

### Bacterial strains

Between 2017 and 2019, clinical samples (*n* = 9) were collected from pigs with signs suggestive of *A. suis* infection in seven commercial farms in western Canada. Isolates were obtained from lungs (*n* = 6), heart (*n* = 2) and brain (*n* = 1) (Table 1). Samples were streaked on 5% blood agar and incubated at 37°C under aerobic conditions and plates were examined at 24 and 48 h for growth. Matrix‐assisted laser desorption/ionisation‐time of flight was performed for initial identification; colonies identified as *A. suis* were selected and saved for further genomic analysis.

**TABLE 1:** Metadata associated with *Actinobacillus suis* western Canadian strains obtained from diseased pigs

Strain ID	Source organ	Postmortem diagnosis	Collection location	Collection date
20_277_1a	Heart	Septicaemia	Farm 6	June 2020
20_377_1a	Brain	Septicaemia	Farm 7	April 2020
17_471	Lung	Bronchopneumonia	Farm 1	September 2017
4286	Lung	Bronchopneumonia	Farm 2	October 2018
19_419	Lung	Bronchopneumonia	Farm 3	September 2019
19_418	Lung	Bronchopneumonia	Farm 3	September 2019
19_298	Heart	Bronchopneumonia/septicaemia	Farm 4	June 2019
18_466	Lung	Bronchointerstitial pneumonia	Farm 5	October 2018
18_292	Lung	Bronchopneumonia	Farm 6	June 2019

### Genome sequencing and assembly

Genomic DNA from isolates was extracted using DNeasy ultraclean microbial kit (QIAGEN, Toronto, Ontario, Canada). Sequencing libraries for isolates 19_419, 19_418, 18_466, 19_298, 18_292, 4286 and 17_417 were prepared using the Nextera XT DNA library (Illumina, San Diego, CA, USA) preparation kit according to the manufacturer's instructions. Library quality was assessed using bioanalyser (Agilent Technologies, Santa Clara, CA, USA) and sequencing was performed using an Illumina MiSeq Platform (Illumina Inc., 500 cycles, 2 × 250) and MiSeq V3 reagent kits (Illumina Inc.). Isolates 20_377_1a and 20_277_1a had DNA libraries prepared using a Rapid Sequencing Kit (SQK‐RAD0003, Oxford Nanopore Technologies, Oxford, UK). Libraries were sequenced on a MinION sequenced (Oxford Nanopore Technologies) using a Flow Cell model FLO‐MIN 106 v R9.4 running the software MinKNOWN v1.10 (Oxford Nanopore Technologies) with default parameters. Genomes were trimmed, assembled, annotated and analysed using the comprehensive genomic analysis tool in Pathosystem Resource Integration Center (PATRIC).^15^

### Phylogenetic tree of *Actinobacillosis suis* and whole genome comparisons

Three complete and four draft *A. suis* genomes isolated from pigs with clinical disease suggestive of *A. suis* infection were included in these analyses, as well as *A. pleuropneumoniae* strain NCTC 10976, which was included in the analysis as an outgroup. These were initially identified between 1963 and 1991 in North America; they were retrieved from National Center for Biotechnology Information (www.ncbi.nlm.nih.gov) (Table 2). Randomised accelerated maximum likelihood for high‐performance computing (RAxML‐VI‐HPC) in PATRIC using 500 genes and allowing neither deletions nor duplications was used to build the phylogenetic tree.^16^ Orthologous average nucleotide identity was performed to measure the overall similarity between genome sequences using OrthoANI V0.93.1 available at EzBioCloud.^17^ Protein sequences of all western Canadian strains were compared to the reference genome ATCC 33415 using bidirectional BLASTP with parameters set to minimum coverage = 30%, minimum identity = 10% and BLAST *E*‐value = 1e‐5 and a circular genomic map was constructed.^15^, ^18^ Progressive Mauve was used to reorder contigs and align query genomes to ATCC 33415 to identify homologous regions distribution.^15^, ^18^

**TABLE 2:** Publicly available *Actinobacillus suis* genomes used for comparison with western Canadian strains

Strain ID	Serovar	Country, province of origin	Isolation source	Collection year	Reference
ATCC 33415	O1:K1	USA	Pig with septicaemia	Not available	19
ATCC 15557	O1:K1	USA, Washington DC	Blood of irradiated adult pig	1963	20
NCTC 12996	Not available	Not available	Pig with septicaemia	1980	NCBI
AS108/13	Not available	Brazil, Minas Gerais	Pig with pneumonia	2013	21
H89‐1173	O2:K3	Canada, Ontario	Lung of pig with pneumonia	1989	22
H91‐0380	O2:K2	Canada, Ontario	Pig with septicaemia	1991	23
H91‐0406	O2:K3	Canada, Ontario	Lung of pig with pneumonia	1991	22

Abbreviation: NCBI, National Center for Biotechnology Information.

### Functional characterisation

Genomes were screened for the presence of virulence factors using BLASTP.^10^, ^11^, ^24^ Antimicrobial resistance genes (ARG) in all *A. suis* genomes were identified using the Resistance Gene Identifier platform and the Comprehensive Antimicrobial Resistance Database, only genes with greater than 80% identity were reported.^15^, ^25^

## RESULTS

### Whole genome comparisons and phylogenetic analysis

**FIGURE 1:**
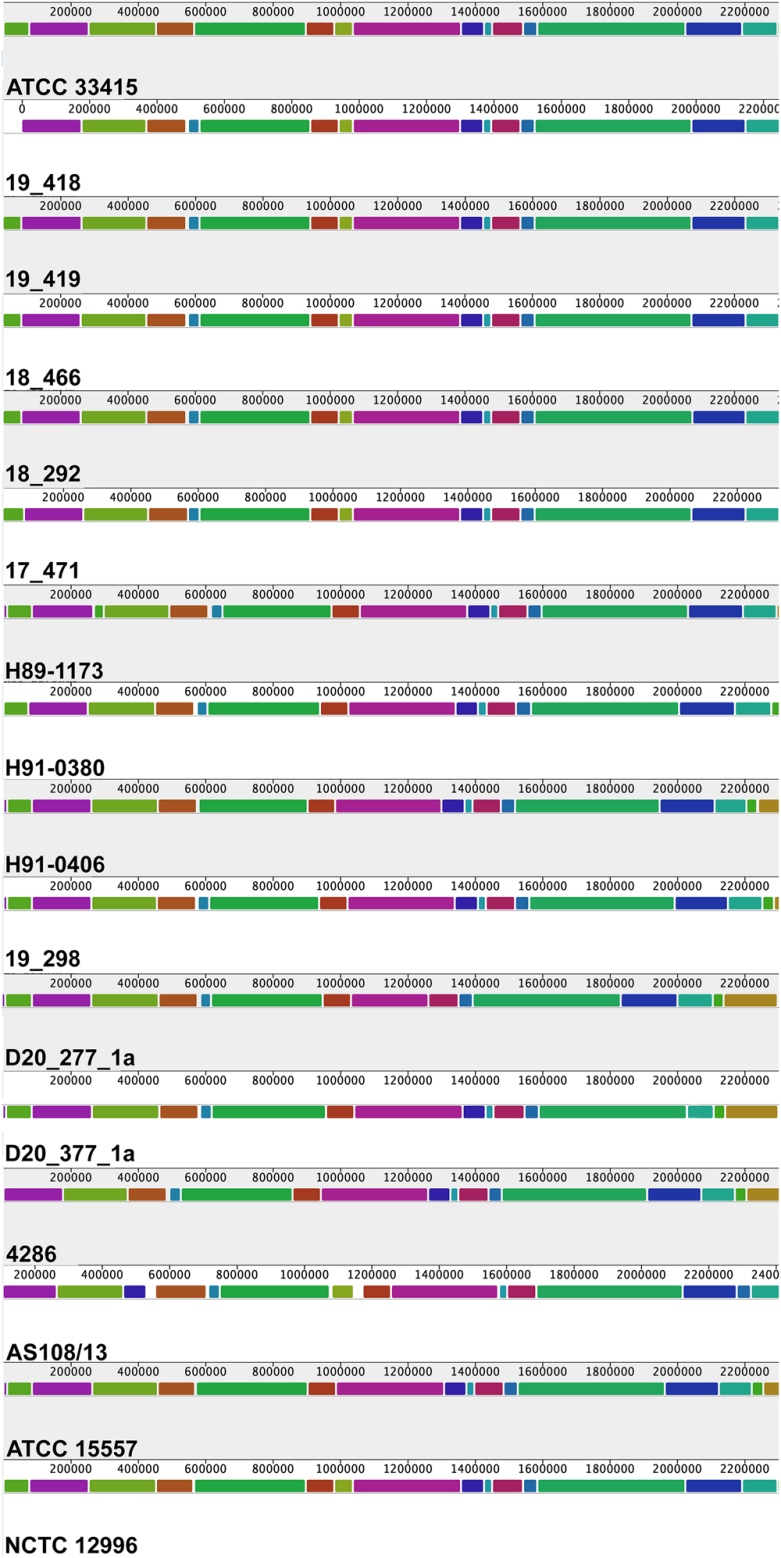
Multiple genome alignment across all strains used in this study. Blocks with the same colours depict homologous regions

A summary of the *A. suis* genomes used in this study is listed in Table 3. Average genomic length and number of genes identified were numerically higher in the seven genomes from this study (2.52 ± 0.4 Mbp and 2478 ± 173 genes) when compared to publicly available genomes (2.48 ± 0.6 Mbp and 2328 ± 107 genes). Average nucleotide identity (ANI) scores for all pairwise comparisons, including previously published genomes, were greater than 99% (Table 4). Noteworthy, isolates 18_466, 18_292, 19_419, 19_418, 17_471, H91‐0406 and H91‐0380 came from different barns and were 99.9% similar to H89‐1173, which was isolated from a diseased pig in Ontario (in 1989). Multiple genome alignment identified a genomic region present in strains ATCC 33415, 17_471 and AS108/13; this was absent in strains 4286, ATCC 15557, H89‐1173, H91‐0380, H91‐0406, 19_298, 20_277_1a and 20_377_1a (Figure 1, yellow region at approximately 1 Mbp). Strain AS108/13 was also noted to have two strain‐specific regions at approximately 0.5 and 1.2 Mbp (Figure 1).

**TABLE 3:** Summary of *Actinobacillus suis* genomes included in in silico analyses

Strain ID	Source	Genome accession	Contigs	GC content (%)	Sequence length (bp)	# Genes identified
20_277_1a	This study	CP090556^a^	2	40.26	2549182	2775
20_377_1a	This study	JAKEZJ000000000	1	40.23	2551416	2772
17_471	This study	SAMN24451496	37	40.08	2533834	2416
4286	This study	JAKEZI000000000	29	40.16	2456581	2320
19_419	This study	JAJUOY000000000	29	40.09	2534652	2414
19_418	This study	SAMN24451500	30	40.09	2534921	2412
19_298	This study	JAJUOZ000000000	29	40.16	2453999	2319
18_466	This study	SAMN24451498	30	40.14	2569370	2467
18_292	This study	JAJUPA000000000	28	40.09	2536093	2416
ATCC 33415	Public	CP009159	1	40.22	2501598	2341
ATCC 15557	Public	NZ_MTBV00000000	80	40.22	2409579	2179
NCTC 12996	Public	NZ_LT906456.1	1	40.22	2501959	2338
H91‐0380	Public	CP003875	2	40.24	2484940	2249
AS108/13	Public	QCXP01	44	40.17	2617798	2528
H89‐1173	Public	NZ_MTBW00000000	74	40.23	2450899	2345
H91‐0460	Public	NZ_MTBX00000000	116	40.35	2423500	2318

^a^ BioProject ID PRJNA792721.

**TABLE 4:** Average nucleotide identity (%) between clinical (this study) and publicly available *Actinobacillus suis* genomes

Strain ID	20_377_1a	18_292	ATCC 33415	ATCC 15557	20_277_1a	NCTC 12996	AS108/13	18_466	17_471	H89‐1173	H91‐0380	12_298	H91‐0460	19_491	19_418	4286
20_377_1a	*	99.78	99.75	99.63	99.83	99.75	99.87	99.78	99.77	99.82	99.81	99.65	99.84	99.78	99.78	99.96
18_292	99.78	*	99.75	99.68	99.79	99.75	99.42	100	99.99	99.93	99.91	99.73	99.92	100	100	99.82
ATCC 33415	99.75	99.75	*	99.74	99.75	100	99.46	99.75	99.74	99.8	99.78	99.58	99.81	99.75	99.75	99.8
ATCC 15557	99.63	99.68	99.74	*	99.69	99.77	99.77	99.69	99.68	99.75	99.72	99.51	99.74	99.69	99.69	99.73
20_277_1a	99.83	99.79	99.75	99.69	*	99.76	99.87	99.8	99.79	99.84	99.83	99.75	99.85	99.8	99.8	99.83
NCTC 12996	99.75	99.75	100	99.77	99.76	*	99.44	99.75	99.75	99.78	99.77	99.57	99.8	99.75	99.75	99.78
AS108/13	99.87	99.42	99.46	99.77	99.87	99.44	*	99.19	99.18	99.89	99.88	99.7	99.9	99.18	99.18	99.91
18_466	99.78	100	99.75	99.69	99.8	99.75	99.19	*	99.97	99.94	99.92	99.73	99.94	99.98	99.98	99.83
17_471	99.77	99.99	99.74	99.68	99.79	99.75	99.18	99.97	*	99.92	99.9	99.71	99.92	100	100	99.82
H89‐1173	99.82	99.93	99.8	99.75	99.84	99.78	99.89	99.94	99.92	*	99.98	99.79	99.99	99.93	99.93	99.89
H91‐0380	99.81	99.91	99.78	99.72	99.83	99.77	99.88	99.92	99.9	99.98	*	99.77	99.98	99.92	99.92	99.87
12_298	99.65	99.73	99.58	99.51	99.75	99.57	99.7	99.73	99.71	99.79	99.77	*	99.78	99.72	99.72	99.68
H91‐0460	99.84	99.92	99.81	99.74	99.85	99.8	99.9	99.94	99.92	99.99	99.98	99.78	*	99.92	99.92	99.88
19_491	99.78	100	99.75	99.69	99.8	99.75	99.18	99.98	100	99.93	99.92	99.72	99.92	*	100	99.84
19_418	99.78	100	99.75	99.69	99.8	99.75	99.18	99.98	100	99.93	99.92	99.72	99.92	100	*	99.84
4286	99.96	99.82	99.8	99.73	99.83	99.78	99.91	99.83	99.82	99.89	99.87	99.68	99.88	99.84	99.84	*

**FIGURE 2:**
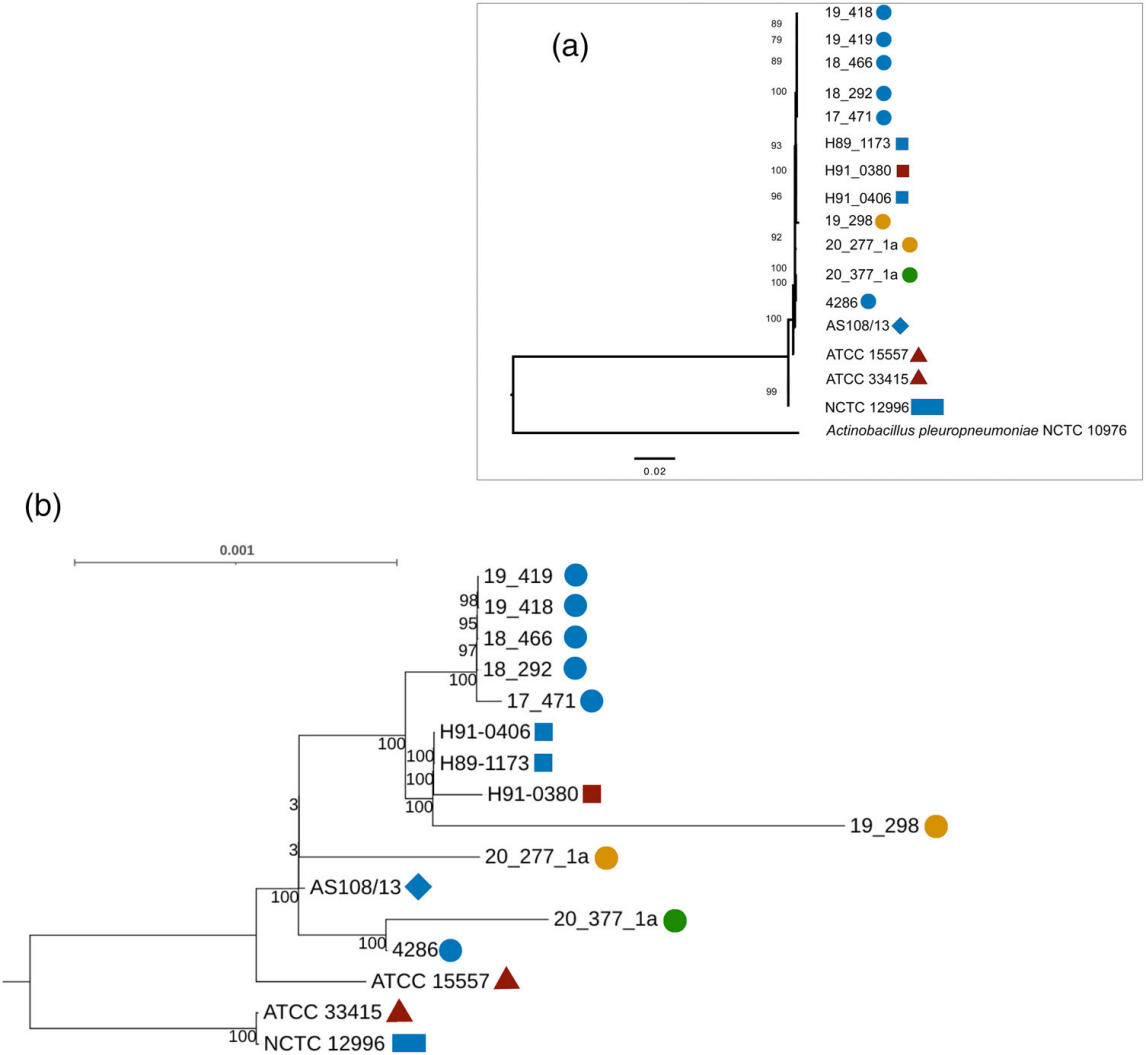
Phylogenetic relationship of *Actinobacillus suis* strains identified in this study (*n* = 9) and publicly available strains (*n* = 7) in relation to a *Actinobacillus pleuropneumoniae* reference strain (a) and the *A. suis* strains only (b). Trees were constructed using RAxML program by comparing 500 randomly selected genes. Organ from which isolates were obtained are coded by colour (lung: blue; heart: orange; blood: red; brain: green). Shapes indicated geographical origin of each isolate (circle: Manitoba, Canada; square: Ontario, Canada; triangle: USA; diamond: Brazil; rectangle: UK)

Phylogenetic analysis (Figure 2a) revealed two groups, one composed of the *A. suis* strains, markedly separated from the *A. pleuropneumoniae* NCTC 10976 strain. When investigating the *A. suis* strains only (Figure 2b), western Canadian isolates obtained from lungs clustered together; they were found closely related to the three reference isolates recovered two decades ago (H91‐0406, H89‐1173, H91‐038) from Ontario, Canada, two of which were also obtained from lungs samples.

While European and USA isolates clustered separately from Canadian *A. suis* isolates, the position of the South American strain AS108/13 is unclear. This strain clustered separately or within the Canadian cluster depending on the phylogenetic reconstruction (Figure 2a,b), sometimes with low bootstrap values. Genome sequences from other South American strains will be necessary to clarify the interrelationship between *A. suis* of the different geographic regions.

### Functional characterisation

**FIGURE 3:**
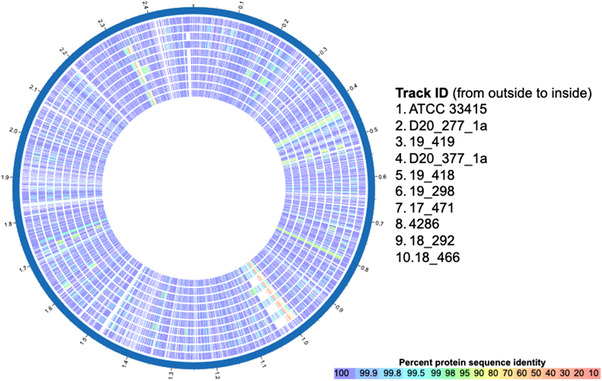
Circle plot depicting protein sequence homology between nine clinical *Actinobacillus suis* isolates relative to the reference strain ATCC 33415

Proteome analysis revealed that most proteins encoded were greater than 99% similar to the reference genomes. However, a few sections with only 20%–70% identity were also identified in all genomes (Figure 3). Genomic gaps in four of nine and seven of nine western Canadian strains, when compared to the reference strain, were identified at 1.0 and 2.5 Mbp regions, respectively. The complete list of protein sequences with less than 70% similarity and gaps where proteins were only identified in ATCC 33415 are provided in Supporting Information S1. In all genomes, five predicted proteins were identified with less than 70% similarity: long‐chain fatty acid transport protein, Cu(l)‐responsive transcriptional regulator, lead, cadmium, zinc and mercury‐transporting ATPase, cell division proteins FtsP and CopG (copper reductase). Strains 20_277_1a, 20_377_1a and 4286 were the only strains with less than 70% similarity to ATCC 33415 KptS gene (capsular polysaccharide ABC transporter). This region also contained a tyrosine recombinase, usually involved in chromosomal integration of mobile genetic elements.

Putative virulence genes were identified in all western Canadian strains (Table 5). Noteworthy, strains 20_277_1a and 20_377_1a, which were isolated from heart and brain samples, carried more RTX‐associated genes (*n* = 9) than all other strains (including ATCC 33415 and all other strains isolated from lung samples). Antimicrobial resistance genes were identified only in western Canadian strains 20_277_1a and 20_377_1a, which had an ARG profile similar to the publicly available genomes (Table 6). The exception was strain AS108/13 that harboured more ARG genes than all other strains, in a unique profile that may be related to the presence of the unique genome regions mentioned above.

**TABLE 5:** Virulence‐associated genes

Virulence factors	Reference	ATCC 33415	20_277_1a	20_377_1a	17_471	4286	19_419	19_418	19_298	18_466	18_292	H91‐0380	H91‐0406	H89‐1173	NCTC 12996	ATCC 15557	AS108/13
Source organ		Blood	Heart	Brain	Lung	Lung	Lung	Lung	Heart	Lung	Lung	‐	Lung	Lung	Blood	Blood	Lung
Autotransporters	42	8	13	11	8	7	7	7	9	8	6	8	8	8	8	8	8
Outer membrane proteins	42	13	12	12	12	12	12	12	12	12	12	13	12	12	13	12	13
Filamentous haemagglutinin	42	2	7	4	1	1	1	1	1	1	1	2	1	1	2	2	2
Sialic acid	44	2	2	2	2	2	2	2	2	2	2	2	2	2	2	2	2
Lipopolysaccharides	44	7	9	9	7	7	7	7	7	7	7	7	7	7	7	7	7
Biofilm	19	4	5	6	4	4	4	4	5	4	4	4	4	4	4	4	4
Iron acquisition	45	28	34	35	28	28	28	28	28	28	28	28	28	28	28	28	28
Repeat in toxins	10	8	9	9	4	4	4	4	4	4	4	4	4	4	8	4	4

**TABLE 6:** Antibiotic resistance genes in clinical *Actinobacillus suis* strains obtained in this study

Strain ID	Resistance genes (>80% identity)
20_277_1a	EF‐Tu
20_377_1a	EF‐Tu
19_419	None
19_418	None
17_471	None
4286	None
18_292	None
18_466	None
19_298	None
ATCC 33415	EF‐Tu
ATCC 15557	EF‐Tu
H89‐1173	EF‐Tu
H91‐0380	EF‐Tu
H91‐0406	EF‐Tu
NCTC 12996	EF‐Tu
AS108/13	ROB‐10, Tet(H), APH(3″)‐IB, APH(6)‐Id, Sul2, EF‐Tu

Abbreviations: APH(3″)‐IB, aminoglycoside; APH(6)‐Id, aminoglycoside; EF‐Tu, *Escherichia coli* EF‐Tu mutants conferring resistance to pulvomycin/elfamycin class; ROB‐10, cephalosporin, penam; Sul2, sulphonamide; Tet(H), tetracyclines.

## DISCUSSION

In this study, we showed that *A. suis* isolates obtained from organs without a mucosal surface harboured an increased number of RTX toxin genes, a potential disease determinant in *A. suis*. We also observed that clinical *A. suis* strains circulating in Canadian commercial herds between 2018 and 2019 were phylogenetically similar to those characterised in the late 1900s. Taken together, these findings reiterate the importance of *A. suis* surveillance to prevent the introduction of new strains and sheds a light on potential markers of virulence.

RTX toxins are widespread in the *Actinobacillus* genus. These toxins have haemolytic, cytotoxic and cytolytic activity; they can induce the production of inflammatory mediators, thereby leading to necrosis in host tissue.^26^ In this study, we found an increased number of RTX‐coding genes in isolates obtained from theoretically sterile organs. In yeast, an increased number of gene copies resulted in increased protein production.^27^ When yeasts were grown under stress, such as glucose‐deprived environment, cells with multiple gene copies associated with glucose metabolism were selected and thrived in that environment.^28^, ^29^ In addition, *E. coli* and *Streptococcus pneumoniae* strains, with increased antimicrobial resistance gene copies, show increased phenotypic resistance to specific drugs.^29^, ^30^ Gene copy number change is a phenomenon established as part of adaptation and evolution of bacteria.^31^ RTX toxins are suggested as disease determinants in *A. suis*.^10^ Previous reports investigating the *A. suis* genomes have reported a maximum of seven RTX gene copies per genome, but no correlation with clinical findings was performed.^32^ We postulate that strains with increased RTX copies may more efficiently colonise non‐mucosal tissues (such as blood) reaching immune‐privileged organs, leading to increased pathogenicity. Given the small number of strains with an increased number of RTX gene copies identified in this study, it remains to be verified if this change truly is associated with increased virulence. Further research focused on evaluating this change using animal models may help clarify this question. Several other virulence factors, such as lipopolysaccharides (LPS), capsular polysaccharides, proteins associated with iron chelation and cellular adhesion also play a role in *A. suis* pathogenesis.^10^, ^33^ Changes in the LPS structure also appear to be associated with the range of virulence observed in different *A. suis* strains.^34^ Adhesins facilitate bacterial colonisation and biofilm formation, including the adhesion of other bacteria.^24^ Finally, this bacterium also carries receptors that recognise and bind to haemoglobin‐bound iron, transferring it to the bacterial cytoplasm.^33^

Despite decades between the isolation of the strains obtained in this study and some of those publicly available, withstanding the exposure to antibiotics used for pork production, we found limited evidence of major changes to the gene content of *A. suis* isolated from diseased pigs. In fact, some of the current strains were phylogenetically very similar those isolated in the last century, with a high nucleotide similarity score. Limited genomic fragment insertions were identified in the western Canadian strains and the gene order identified was the same. It has been established that the genome structure of bacteria can change slowly with time due to local or global mutations.^35^, ^36^, ^37^

Treatment and control of *A. suis* is usually achieved through antimicrobial therapy, often based on ceftiofur, gentamicin and trimethoprim/sulphadiazine, ampicillin, sulphadimethoxine or tiamulin.^38^ Interestingly, we did not identify ARG related to any drugs used in pork production in the western Canadian strains. The only strain harbouring ARG for β‐lactams, sulphadiazine and sulphadimethoxine was AS108/13. One caveat in this analysis was the lack of phenotypic antimicrobial susceptibility data. While future investigations should focus on evaluating the efficacy of *A. suis* whole genome sequencing in predicting resistance patterns, it has been shown to be highly correlated to phenotypical resistance results for other bacterial species.^39^ Despite this, we did identify nucleotides changes in the gene coding for the copper resistance protein CopG present in the western Canadian strains, when compared to the ATCC 33415 reference strain. CopG encodes a 14 kDa periplasmic protein that is suggested to contribute to copper resistance by reducing free copper ions.^40^ This protein is widely spread across Gram‐negative bacterial species; genes encoding CopG homologues are often found associated with other known metalloproteins.^40^ One study^41^ proposed that CopG is the main copper detoxification mechanism employed by *Vibrio cholerae* when under anaerobic conditions. Future research may further explore how the changes identified in the western Canadian strains contribute to the biological activity of CopG.

*Actinobacillus suis* remains a challenge for swine production. As the livestock industry moves towards a reduction in overall antimicrobial usage, it is likely that this agent will become more relevant in the coming years. Here, we have shown that isolates obtained from brain and heart had more RTX toxin gene copies in their genomes than those isolated from lungs. We have shown that *A. suis* recently circulating in commercial farms in Canada are similar to isolates from 1990 to 2000s. Antimicrobial resistance genes detected in clinical isolates suggest the potential for resistance to drugs of importance to human medicine.

## AUTHOR CONTRIBUTIONS

Matheus de Oliveira Costa acquired funding and conceptualised the study. Matheus de Oliveira Costa, Dharmasiri Gamage Ruwini Sulochana Kulathunga and Alaa Abou Fakher performed the analyses. Dharmasiri Gamage Ruwini Sulochana Kulathunga drafted the first version of the manuscript. Matheus de Oliveira Costa reviewed the manuscript and the data analyses.

## CONFLICTS OF INTEREST

The authors declare they have no conflicts of interest.

## ETHICS STATEMENT

No ethical approval was required because the work did not directly use any live pigs.

## Supporting information

Supporting information

Supporting information

## Data Availability

Supporting information data are available at https://doi.org/10.23644/uu.19346372
